# Effect of Unit Cell Shape on Switchable Infrared Metamaterial VO_2_ Absorbers/Emitters

**DOI:** 10.34133/2021/9804183

**Published:** 2021-04-22

**Authors:** Feifei Ren, Jinxin Gu, Hang Wei, Gaoping Xu, Jiupeng Zhao, Shuliang Dou, Yao Li

**Affiliations:** ^1^Center for Composite Materials and Structure, Harbin Institute of Technology, Harbin 150001, China; ^2^School of Chemistry and Chemical Engineering, Harbin Institute of Technology, Harbin 150001, China

## Abstract

Metamaterial absorber/emitter is an important aspect of infrared radiation manipulation. In this paper, we proposed four simple switchable infrared metamaterial absorbers/emitters with Ag/VO_2_ disks on the Ag plane employing triangle, square, hexagon, and circle unit cells. The spectral absorption peaks whose intensities are above 0.99 occur at ~4 *μ*m after structure optimization when VO_2_ is in insulating state and disappear when VO_2_ becomes metallic state. The simulated electromagnetic field reveals that the spectral absorption peaks are attributed to the excitation of magnetic polariton within the insulating VO_2_ spacer layer, whose values exceed 1.59 orders of magnitude higher than the incident magnetic field. Longer resonant wavelength would be excited in square arrays because its configuration is a better carrier of charges at the same spans. For absorption stability, the absorbers/emitters with square and circular structures do not have any change with the polarization angles changing from 0° to 90°, due to the high rotational symmetric structure. And four absorbers/emitters reveal similar shifts and attenuations under different incident angles. We believed that the switchable absorber/emitter demonstrates promising applications in the sensing technology and adaptive infrared system.

## 1. Introduction

An object would absorb radiation from surrounding space and reradiates internal energy to outside, and the values of that are determined by its temperature and electromagnetic (EM) wave frequency, as described by Planck's law [1]. For infrared detection, the existence of the atmospheric transparent window of 3-5 *μ*m and 8-14 *μ*m limits the EM waveband of infrared detection, the rest of which would be mainly absorbed heavily by CO_2_ and water in the air [2, 3]. The detectable radiation control has long been a research topic of interest for scientists [4, 5]. Emission bands whose radiation is much narrower are suitable for various scenarios [6, 7]. For example, Zhu et al. designed thermal management devices by combining wavelength-selective emitters and thermal insulators and achieved emittance of ~0.58 and~0.08 within 5-8 and 8-14 *μ*m, reducing the surface temperature of a high-temperature object (873 K) to 410 K [8].

An alternative approach to achieve selective emitters is metamaterial absorbers/emitters, which possess high absorptance at specific wavelengths, and it has been attracted great attention for radiation regulation applications. An acceptable explanation of perfect absorption is the impedance of metamaterial that would be equivalent to the outside. It means perfect absorption could be achieved through extraordinary structural design for a specific frequency [9–11]. The metamaterial absorbers/emitters above mentioned have extensive applications [12]. For example, the working wavelengths absorptance of sensing device needs to be as high as possible to weaken noise influences [13, 14]. Liu. et al. experimentally realized a narrow band mid-infrared thermal emitter with cross-shaped resonator, which is also capable of engineering the emittance over a large bandwidth in the desired wavelength-dependent manner [15]. However, most of the metamaterial absorbers/emitters are fixed once the structure is fabricated. Of these, the phase-change materials, such as VO_2_ and Ge_2_Sb_2_Te_5_ (GST) whose permittivity changes dramatically with stimulation [16–19], were introduced to metamaterial absorbers/emitters to obtain the function of manipulating emittance or infrared radiation [12, 20–22]. Cao et al. designed an ultrathin reconfigurable metasurface based on Au_/_SiO_2_/Ge_2_Sb_2_Te_5_/Au multilayer and realized dynamic control of multispectral thermal emission from 2 to 4 *μ*m [22]. Long et al. prepared a metamaterial with aluminum submicron disks and phase-change VO_2_ spacer on a metal ground plane and exhibited an absorption peak close to unity at 7 *μ*m at room temperature as a selective absorber [23]. Sun et al. developed a VO_2_-based metamaterial emitter which radiates light in both 3-5 *μ*m and 8-14 *μ*m atmospheric windows at room temperature. At high temperatures, the radiation peaks move out of the atmospheric windows and results in strong radiation at 5-8 *μ*m [24]. The principles of the aforementioned spectral absorption peak are the excitation of magnetic polariton or standing waves or other kinds of resonances [21, 25–30]. Although the abovementioned reversible selective metamaterial absorbers have been manufactured, and the roles and influences of unit cell shapes on metamaterial in forming selective absorption peaks have been barely reported until now.

In this paper, four simple switchable infrared metamaterial absorbers/emitters with Ag/VO_2_ disks on the Ag plane by magnetic polariton employing triangular, square, hexagonal, and circular unit structure are proposed. The formation mechanisms of absorption peak, as well as absorption stability on different shapes of the unit structure, are investigated innovatively. It is believed that switchable absorbers/emitters provide promising applications in adaptive infrared technology.

## 2. Experimental Section

Numerical simulation based on the finite-difference time-domain (FDTD) was employed to calculate the spectral directional reflectance. A unit cell with a domain size of 1 *μ*m × 1 *μ*m × 3 *μ*m was simulated. Periodic boundary condition was used at *x* and *y* directions at normal incidence, while Bloch boundary condition was implemented at oblique incidence waves.

The emittance of the object is equal to its absorptance based on Kirchhoff's law at the same temperature as *ε* = *A* = 1 − *R* − *T*, where *ε*, *A*, *R*, and *T* are emittance, absorptance, reflectance, and transmittance of the emitters, respectively. In our case, EM waves cannot pass through the absorbers/emitters. The description of absorbers/emitters is simplified as emitters. The optical constants of Ag were taken from material databases (Palik), and that of both insulating and metallic VO_2_ were obtained from fitted experimental data in references [31, 32].

### 2.1. Design of Switchable Infrared Metamaterial Emitters

**Figure 1:**
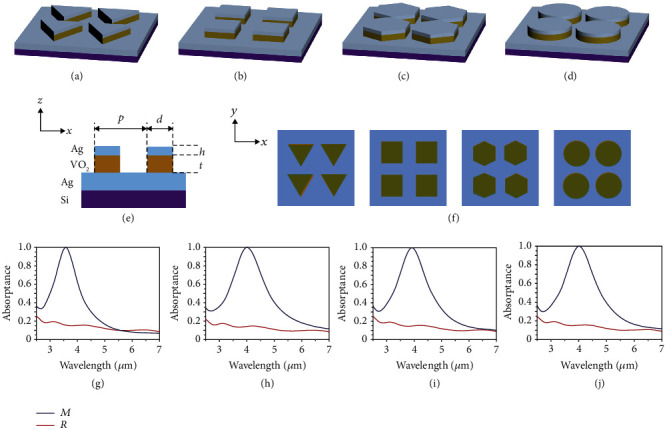
Four switchable infrared metamaterial emitters and their “switching on/off” ability of absorption. (a)–(f) Schematic of four switchable infrared metamaterial emitters consists of Ag/VO_2_/Ag. (g)–(j) The spectral absorptance of emitters employing triangular, square, hexagonal, and circular structures, within 2.5-7 *μ*m when VO_2_ is in insulating (blue lines) and metallic (red lines).

The thermochromic vanadium dioxide (VO_2_) has been placed on forestage of many reversible materials, due to its discrepant reflection and transmittance of films [16, 33, 34]. The concepts of four switchable infrared metamaterial emitters based on VO_2_ are presented in Figures 1(a)–(d). In the unit cell configuration in Figure 1(e), the switchable emitters consist of Ag layer with thickness *h* = 80 nm and VO_2_ layer with thickness *t* = 100 nm on the Ag plane, which forms a typical metal-insulator-metal structure. The patch arrays of Ag and VO_2_ have a period of *p* = 1 *μ*m, and the shapes of that are set as triangle, square, hexagon, and circle, at *x-y* or top views in Figure 1(f). The detailed geometrical parameters of the unit structure are in Fig S1a-d. The absorption peaks of all emitters are optimized and close to unity 4 *μ*m when the temperature is below phase-transition temperature (*T*_*t*_) and disappear when the temperature is above *T*_*t*_. The simulated spectral absorptance under normal incidence is illustrated in Figures 1(g)–(j) for emitters employing triangular, square, hexagonal, and circular structures, respectively. The blue and red solid lines demonstrate the absorptances of four switchable emitters when VO_2_ is in insulating (VO_2_(*M*)) and metallic state (VO_2_(*R*)).

The spans of unit structure for emitters with triangular structure at the *X* and *Y* axes are 980 nm, the absorption peak is located at 3.57 *μ*m when VO_2_ is in insulating state, and the value of that is above 0.998. The spans of the unit structure at the *X* and *Y* axes are 850 nm for emitters with the square structure, the absorption peak is located at 4.02 *μ*m, and the value of that is above 0.999. For emitters with the hexagonal structure, the unit spans at the *X* and *Y* axes are 900 nm, and the absorption peak is located at 3.92 *μ*m, whose value reaches up to 0.998. For emitters with cylinders, the diameter of the cell is 940 nm, the absorption peak is located at 4.02 *μ*m, whose value reaches up to 0.998. But when the VO_2_ becomes metallic state, the absorptances of all emitters drop significantly, presenting the scenario of “switch-off” and show absorptance of 0.15, 0.14, 0.15, and 0.15 for four emitters at associated wavelengths.

## 3. Results and Discussion

### 3.1. Numerical Simulation and LC Circuit Model

**Figure 2:**
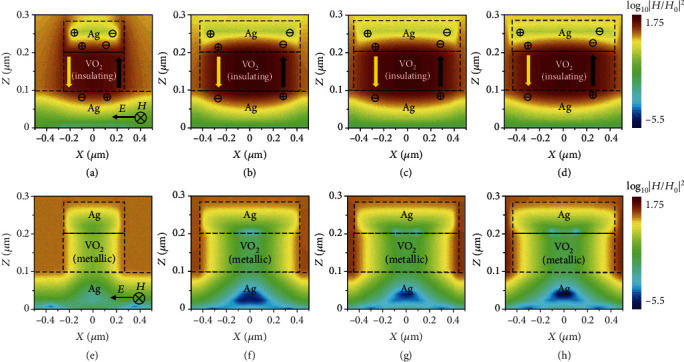
The electromagnetic field distribution when VO_2_ is in insulating state with (a)–(d) *x-z* views crosses the center VO_2_ of triangle, square, hexagon, and circle cell at the wavelength where the absorption peak occurs. (e)–(h) *x-z* views of the electromagnetic field distribution of emitters when VO_2_ is metallic at the same wavelengths.

Generally, the mechanisms of spectral absorption peaks are the excitation of magnetic polariton (MP) or standing waves, or other kinds of resonances. In our case, we focus on MP in insulating VO_2_ between the upper patches of Ag and the lower Ag plane when VO_2_ is in insulating state, which frequently occurred within a metal-insulator-metal structure. The magnetic and electric field distribution within a unit cell at the wavelength of absorption peaks for four switchable emitters ìs in Figure 2, where the squared magnetic field normalized to the incident magnetic field is represented by contours, and the electric field indicates by charge distribution and yellow and black arrows in Ag and VO_2_ layers. Four *x-z* cross-section views at the center for switching emitters with triangular, square, hexagonal, and circular structures, respectively, are studied in Figures 2(a)–(d) and 2(e)–(h). For the insulating VO_2_, when a time-varying electric field along the *x* axis is applied to patches of upper Ag, the interfaces between upper Ag patches and VO_2_ or air would generate a time-varying amount of positive or negative charges. At the same time, the bottom Ag plane would generate charges of opposite signs comparing with the upper interface at one moment. For instance, when the electric field along the *x* axis directions goes from negative to zero, negative and positive charges at the right and left edges of the patch of Ag would accumulate as charge shown in Figures 2(a)–(d), and charges with opposite signs accumulate at the bottom Ag plane, and the electric fields with opposite directions generate on two sides of the VO_2_ layer. The opposite electric fields reverse their direction periodically as the external EM wave arrives periodically, leading to a time-varying oscillating electric field in the insulating VO_2_ layer. Consequently, the MP is excited between the time-varying incident EM waves and induced magnetic fields by oscillating electric field inside the insulator at a specific frequency, resulting in an absorption enhancement.

From simulated magnetic field at the absorption peak for the case of insulating VO_2_, the enhanced magnetic fields are confined within the VO_2_ layer, which are 1.79, 1.59, 1.68, and 1.64 orders of magnitude higher than the incident magnetic field. However, the patches of upper Ag and VO_2_ are integrated with the bottom Ag layer when the VO_2_ becomes metallic in Figures 2(e)–(h). The electric field would not exist between center dotted lines, leading to the disappearing of MP. The switching emitters can be actuated by VO_2_ phase transition.

**Figure 3:**
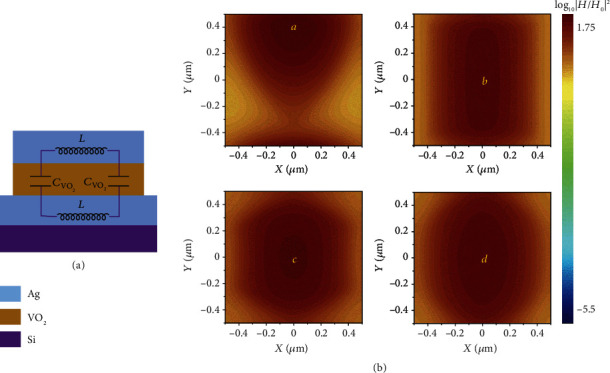
(a) The inductor-capacitor (LC) circuit consists of capacitors and inductors, which will resonate at MP frequency. The imaginary part of impedance at the resonant frequency needs to become zero if the condition of perfect absorption is achieved. (b) The magnetic field distribution when VO_2_ is in insulating state at *x-y* views crosses the center VO_2_ of triangle, square, hexagon, and circle cell at resonant wavelengths.

An equivalent LC circuit model can be used to analyze the excitation of MP in Figure 3(a). To simplify the calculation process, we calculate the magnetic resonance frequency by the emitter with the square structure. In our case, the insulating VO_2_ patches and paralleled Ag layers can be regarded as capacitors, while the patches of the upper Ag and bottom Ag plane serve as inductors. The charges across the capacitor would accumulate on the upper and bottom interface. A vectorial electrical current loop generates in closed circuit including components of two capacitors and inductors. It is obvious that the imaginary part of impedance at the resonant frequency needs to become zero (the resistance did not exist) if the condition of perfect absorption is achieved [23, 25, 26, 35].

The imaginary part impedance of the circuit can be calculated by

(1) $$\begin{array}{c} Z_{\text{ima}}=2\left(\frac{1}{i\omega C_{\text{v}{\text{o}}_{2}}}+i\omega L_{\text{Ag}}\right), \end{array}$$

where *w* is the angular frequency. The capacitance of the insulating *C*_VO2_ can be determined from the parallel plate as the following:

(2) $$\begin{array}{c} C_{\text{v}{\text{o}}_{2}}=\frac{c_{1}\varepsilon_{0}\varepsilon_{r}S}{t}, \end{array}$$

where *c*_1_ is the coefficient accounting for the nonuniform charge distribution at the Ag surfaces, the value is ~0.2. *ε*_0_, and *ε_r_* is the real part of the permittivity of vacuum and insulating VO_2,_ whose values are 8.85 × 10^−12^ F/m and 2.42. *t* is 100 nm for this case. *S* is the area of Ag cell, the value of which is 7.23 × 10^−14^ m^2^. Therefore, *C*_VO2_ can be estimated to be 3.10 × 10^−17^ F for the square structure.

The inductance of the Ag patch and Ag plane includes the mutual inductance *L*_*m*_ and the kinetic inductance *L*_*k*_, and the former can be calculated by an experiential formula as the following:

(3) $$\begin{array}{c} L_{m}=\frac{k\mu_{0}N^{2}S}{l}=\frac{0.5\mu_{0}tw}{l}, \end{array}$$

where *k*, *μ*_0_, *N*, *S*, *w*, and *l* are coefficients which depend on the ratio between diameter and length of the structure, permeability (4*π* × 10^−7^ H/m), number of loops, the cross-sectional area of current, and width (*x*-direction) and length (*y*-direction) of the structure. Apparently, *L*_*m*_ can be estimated to be about 6.28 × 10^−14^ H for the emitter with the square unit.

Also, the kinetic inductance *L*_*k*_ cannot be ignored because of drifting electrons in nanoscale dimension which can be described as the following:

(4) $$\begin{array}{c} L_{k}=-\frac{w}{\omega^{2}\varepsilon_{0}\varepsilon_{\text{Ag}}\delta_{\text{Ag}}l}, \end{array}$$

where *ε*_Ag_, *δ*_Ag_, *w*, and *l* are the real part of the permittivity, penetration depth of bulk Ag, width, and length of the area where charges accumulate. Noted that the *ε*_Ag_ and *δ*_Ag_ are the functions of angular frequency *ω*. In order to simplify the calculation process, the above two parameters are determined as constants by their simulated resonant wavelength aforehand. For the emitter with the square structure, *w* = *l* = *d* = 8.5 × 10^−7^ m, *ε*_Ag_, and *δ*_Ag_ are about ~-800 and 7.39 × 10^−9^ m, and *L*_*k*_ can be estimated to be about 4.58 × 10^−14^ H by setting *w*^2^*LC* = 1, from formulas (1)–(4). The resonance wavelength can be obtained from the equation as follows:

(5) $$\begin{array}{c} \lambda =\frac{2\pi c_{0}}{\omega }=2\pi c_{0}\sqrt{C_{\text{V}{\text{O}}_{2}}\left(L_{m}+L_{k}\right)}, \end{array}$$

where *c*_0_ is the speed of light in vacuum, and the resonance wavelength calculated is about 4.16 *μ*m for emitter with square cell in insulating VO_2_, which is within a 3% relative error compared to the simulation. It is noted that the capacitances of emitters would increase with the span of unit cell increasing, while inductances of that are functions of widths and lengths. Figure 3(b) shows that coupled magnetic field distributions of four absorbers/emitters are coincident with the above conclusion, in which the highest intensity (points *a*, *b*, *c*, *d*) is located at *x* = 0 *μ*m, *y* = 0.385 *μ*m for the triangular structure, at *x* = 0 m, and *y* = 0 *μ*m for other three cases, which are near the center of maximal spans along with *x*-directions. Comparatively speaking, the emitter with the square unit structure produces maximal capacitances and inductances on the same span within a single period for four cases, which leads to a longer resonance wavelength.

### 3.2. Absorption Peak Locations, Polarization, and Angle Dependency

**Figure 4:**
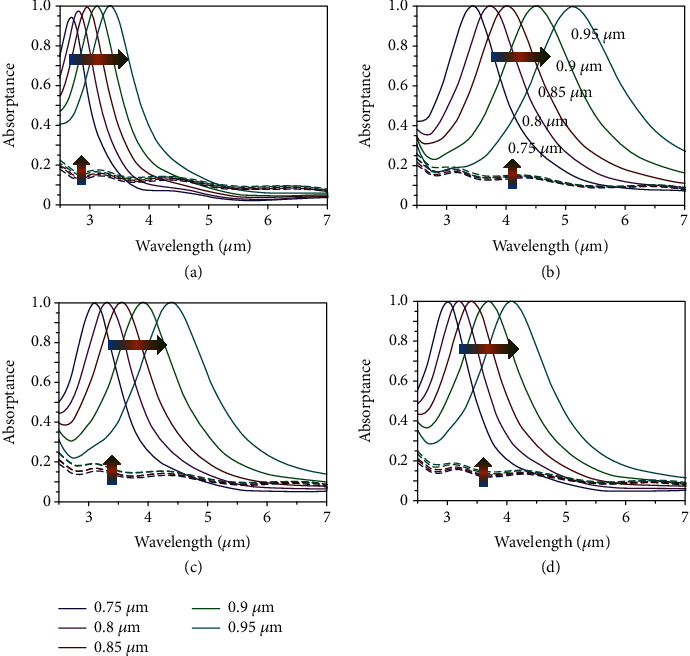
Spectral absorptance under normal incidence with the span of unit cell of Ag and VO_2_ from 0.75 to 0.95 *μ*m for emitters with triangular (a), square (b), hexagonal (c), and circular unit cell (d).

From above, it is implied that the magnetic response of emitter largely depends on the structure geometry of Ag and insulating VO_2_. We investigate the spectral absorptances under normal incidence with the spans of the unit structure (*d*) of Ag and VO_2_ from 0.75 to 0.95 *μ*m for emitters with triangular, square, hexagonal, and circular structures, and results are shown in Figures 4(a)–(d). The switchable property of absorption peaks still works when VO_2_ states transform between insulating and metallic. For the case of insulating VO_2_, the locations of absorption peaks are redshift with the spans of unit cell increase for all emitters. For instance, the *λ*_triangle_ varies from 2.70 to 3.35 *μ*m, and the absorption of that is from 0.94 to 1. In contrast, *λ*_square_, *λ*_hexagon_, and *λ*_circle_ are from 3.44, 3.1, and 3.01 *μ*m to 5.12, 4.38, and 4.08 *μ*m, respectively, and the absorptances of all emitters are above 0.99. However, the absorptances of all emitters are almost unchanged when VO_2_ is in metallic state as shown in the corresponding dotted line. The aforementioned results show that the structure geometry and the area of the unit structure have great influences on the position of resonance frequency from the equivalent LC circuit model.

**Figure 5:**
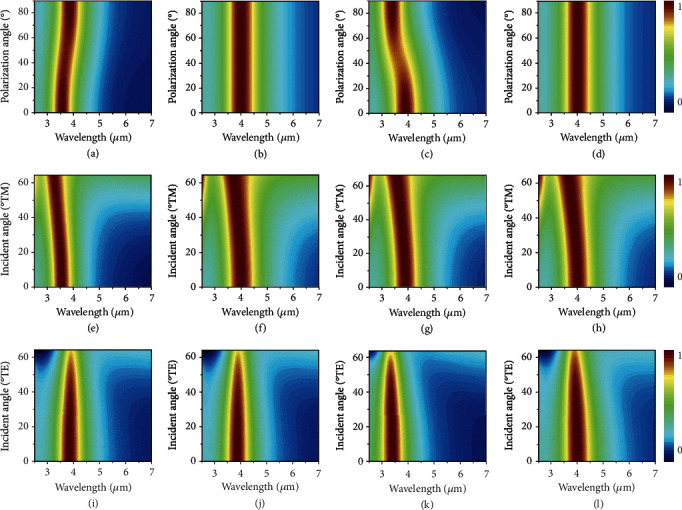
Spectral absorptance of emitters with triangular (a), square (b), hexagonal (c), and circular (d) arrays under the changes in the polarization under normal incidence. Spectral absorptance of emitters under the different incident angles from 0-65° of transverse magnetic (e)–(h) and transverse electric (i)–(l) waves.

The absorption stability of the structure under the changes in the polarization was studied as well. Figures 5(a)–(d) demonstrated correlation of absorptance of four emitters in the region of 2.5-7 *μ*m associated with polarized EM waves under the normal incidence. It can be clearly observed that an extremely high absorption appears around 4 *μ*m for the four emitters. For the emitters with square and circular structures, the absorption peaks do not have any change with the polarization angles changing from 0° to 90°, which indicate the independence of the polarization due to high rotational symmetric structure. But in cases of triangular and hexagonal structures, the locations of absorption peak would redshift to 3.87 *μ*m and blueshift to 3.41 *μ*m, which are within 8.4% and 13% relative error compared to the initial resonant wavelength.

As an absorber or emitter, it is important to be diffuse to absorb radiation from all directions or emit radiation to all directions in particular for energy applications such as radiative thermal management. We investigated the absorption stability of the absorbers under different incident angles from 0 to 65° of transverse magnetic (TM) and transverse electric (TE) polarized wave as well in Figures 5(e)–(i). For cases of incident TM, the locations of all absorption peaks would blueshift to 3.27, 3.75, 3.57, and 3.66 *μ*m at an incident angle of 65°. This is because that the intensity and distribution of oscillating electric field are correlated with an oblique angle of incident TM waves. But for the incident TE waves, in which the intensity and distribution of oscillating electric field are independent of incident angle, the positions of absorption peaks are almost the same and remain close to 4 *μ*m. It is noted that the absorptions would decrease to 0.77, 0.79, 0.71, and 0.81 for the four emitters and produce attenuations of 23%, 21%, 29%, and 19% at the oblique angle of 65°, which exhibit considerable adaptability.

## 4. Conclusion

In conclusion, we proposed and characterized four simple switchable infrared metamaterial absorbers/emitters with Ag/VO_2_ disks on the Ag plane employing triangle, square, hexagon, and circle unit structures. When VO_2_ is in insulating state, the absorbers/emitters exhibit the absorption peaks close to unity 4 *μ*m whose absorptions are above 0.99, but absorption peaks would disappear when VO_2_ becomes metallic state, whose absorptions are as low as ~0.15 and reveal a function of “switch on/off.” The switchable mechanism is contributed to the excitation and vanishment of MP in the VO_2_ layer within Ag. It is noted that square arrays tend to produce longer resonant wavelengths because its configuration is a better carrier of charges. Comparing to the triangle, hexagon structures, the absorbers/emitters with square and circle structures show excellent stability of absorption under the changes in the polarized waves due to their high rotational symmetry in *x-y* cross-section. For the absorption stability under different incident angles, four absorbers/emitters reveal similar shifts and attenuations. It is believed that switchable metamaterial absorbers/emitters could facilitate applications in the dynamic infrared system and thermal detectors.

## Data Availability

“Effect of Unit Cell Shape on Switchable Infrared Metamaterial VO_2_ Absorbers/Emitters” (RESEARCH-D-20-00296): all data [.opj] used to support the findings of this study are available from the corresponding author upon request. The data includes absorptances and electric and magnetic fields distributions of metamaterials designed in this paper.

## Supplementary Materials

Supplementary Materials Figure S1: sizes of Ag/VO2 patches of absorbers/emitters with triangle, square, hexagon, and circle arrays.
