# Supplementary material for: Effect of Unit Cell Shape on Switchable Infrared Metamaterial VO2 Absorbers/Emitters
Source: Research (Wash D C). 2021 Apr 22;2021:9804183. doi: 10.34133/2021/9804183 (PMC8087995; doi:10.34133/2021/9804183)
Supplement: Supplementary Materials — Figure S1: sizes of Ag/VO2 patches of absorbers/emitters with triangle, square, hexagon, and circle arrays. [file 9804183.f1.zip › supporting information.docx]

***Supporting information for***

**Effect of Unit Cell Shape on Switchable Infrared Metamaterial VO_2_ Absorbers/Emitters**

**Feifei Ren,^1^ Jinxin Gu,^2^ Hang Wei,^1^ Gaoping Xu,^1^ Jiupeng Zhao,^2^ Shuliang Dou,^1,*^ Yao Li^1,*^**

*^1^Center for Composite Materials and Structure, Harbin Institute of Technology, Harbin, 150001, P. R. China*

*^2^School of Chemistry and Chemical Engineering, Harbin Institute of Technology, Harbin, 150001, P. R. China*

^*^Corresponding authors: [yaoli@hit.edu.cn](mailto:yaoli@hit.edu.cn), [dousl@hit.edu.cn](mailto:dousl@hit.edu.cn)

**Detailed morphology of four metamaterial absorbers/emitters**

In order to facilitate the analysis of different shape structure, spans of single structure in X and Y directions are set to be same for all absorbers/emitters.

**
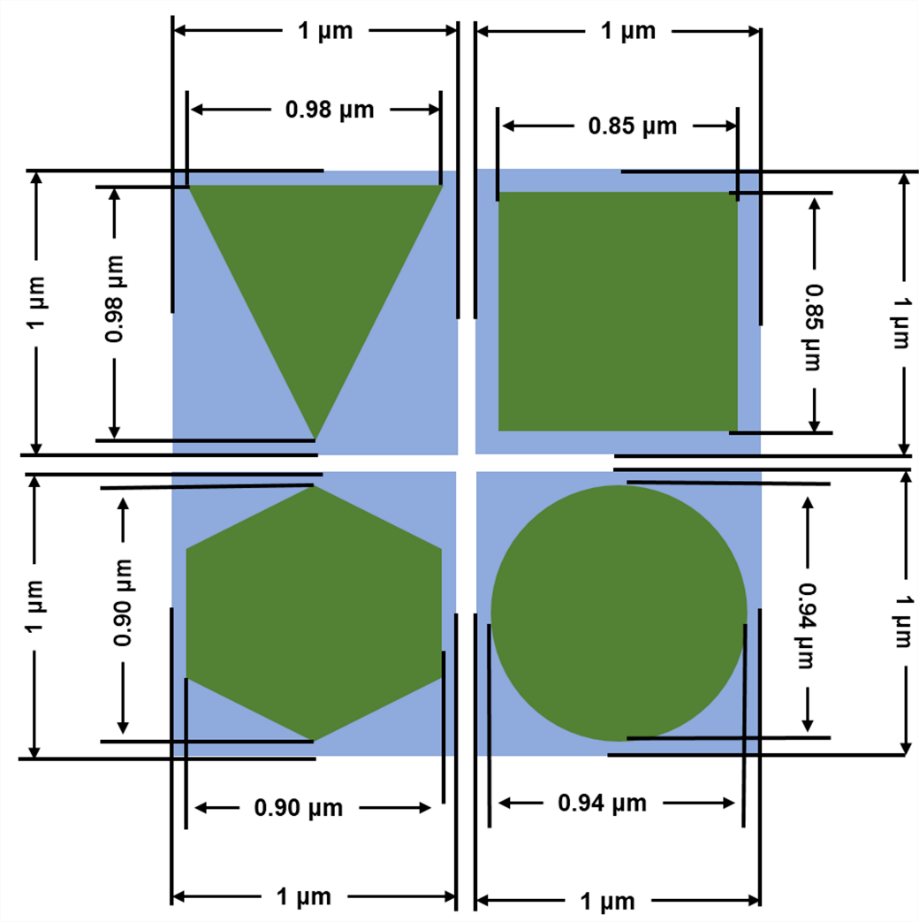
**

**Fig. S1. Sizes of Ag/VO_2_ patches of absorbers/emitters with triangle, square, hexagon and circle arrays**
