# Supplementary figures and images for: Effect of Unit Cell Shape on Switchable Infrared Metamaterial VO2 Absorbers/Emitters
Source: Research (Wash D C). 2021 Apr 22;2021:9804183. doi: 10.34133/2021/9804183 (PMC8087995; doi:10.34133/2021/9804183)

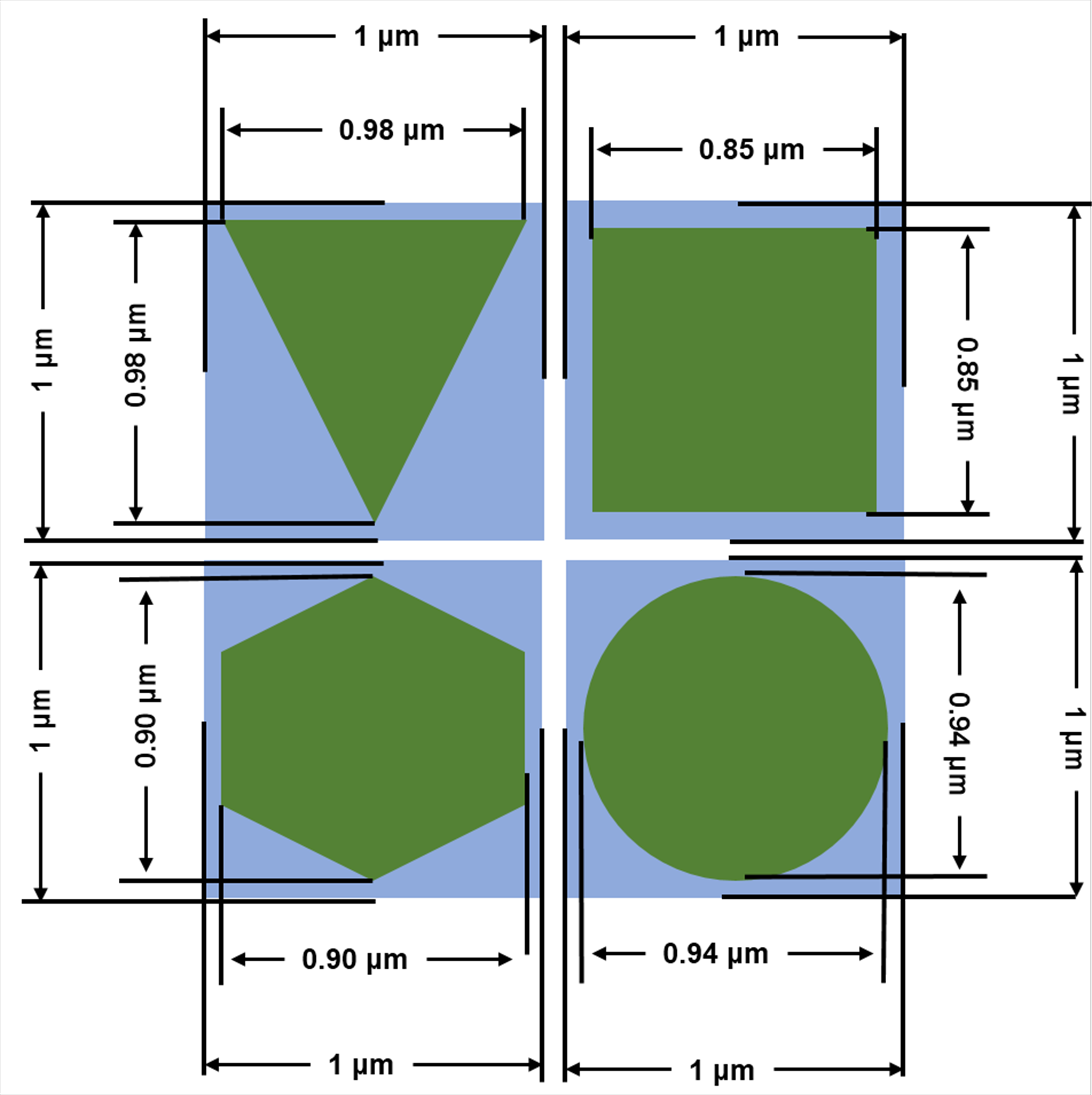

Supplement: Supplementary Materials — Figure S1: sizes of Ag/VO2 patches of absorbers/emitters with triangle, square, hexagon, and circle arrays. [file 9804183.f1.zip › figureS1.tif]
